# Soy Isoflavone Extract Does Not Increase the Intoxicating Effects of Acute Alcohol Ingestion in Human Volunteers

**DOI:** 10.3389/fphar.2019.00131

**Published:** 2019-02-27

**Authors:** Roser Martínez-Riera, Clara Pérez-Mañá, Esther Papaseit, Francina Fonseca, Rafael de la Torre, Nieves Pizarro, Marta Torrens, Magí Farré

**Affiliations:** ^1^Institut de Neuropsiquiatria i Addiccions, Institut Hospital del Mar d’Investigacions Mèdiques (IMIM), Barcelona, Spain; ^2^Department of Psychiatry and Legal Medicine and Department of Pharmacology, Therapeutics and Toxicoloy, Universitat Autònoma de Barcelona, Cerdanyola del Vallés, Spain; ^3^Red de Salud Mental Gipuzkoa, Osakidetza, San Sebastián-Donostia, Spain; ^4^Clinical Pharmacology Unit, Hospital Universitari Germans Trias i Pujol and Institut de Recerca Germans Trias i Pujol (IGTP), Badalona, Spain; ^5^Integrative Pharmacology and Systems Neurosciences Research Group, Institut Hospital del Mar d’Investigacions Mèdiques (IMIM), Barcelona, Spain; ^6^Department of Experimental and Health Sciences (CEXS), Universitat Pompeu Fabra, Barcelona, Spain

**Keywords:** soy extracts, isoflavones, daidzein, genistein, aldehyde-dehydrogenase-2 enzyme, alcohol, clinical trial

## Abstract

Soy beans contain isoflavones, including daidzein and genistein, with biological activities related to therapeutic effects in reducing osteoporosis, decreasing adverse menopausal manifestations, providing protection from cardiovascular diseases, and reducing hormone-dependent cancers and age-related cognitive-decline. Daidzein has been described as inhibiting the aldehyde-dehydrogenase-2 enzyme (ALDH2), and reducing alcohol use in clinical pilot studies. Our aim was to evaluate the possible interactions between a soy extract product and alcohol in a crossover, single blind, randomized study. Ten healthy male volunteers participated in two experimental sessions: one with a single dose of alcohol (0.5 g/kg, Vodka Absolut, Sweden), and the other with four capsules of a soy extract product (Super-Absorbable Soy Isoflavones, Life-Extension, United States) and, 2 h later, the same dose of alcohol. Results showed no differences in vital signs except a slightly higher significative reduction in diastolic blood pressure at 2, 3, 4, and 8 h after administration with alcohol alone in comparison with soy extract+alcohol. Ethanol-induced subjective and adverse effects were similar for both conditions with the exception of headache (higher at 8 h after alcohol alone). Our results demonstrate that a single dose of a soy isoflavone extract did not influence alcohol pharmacokinetics and pharmacological effects and did not induce any disulfiram-reaction symptoms. Soy extract and alcohol did not interact and can be administered safely.

## Introduction

Isoflavones are biologically active polyphenols found in soybeans and other legumes. In the former, the principal ones are daidzin (40%) and genistin (50%) which transform into two active compounds, daidzein and genistein, respectively (U.S. Department of Agriculture, Agricultural Research Service, 2015). Daidzein is also metabolized by intestinal bacteria to the active compound equol. Daidzein consumption has been related to phytoestrogen and antioxidant activity, a lower incidence of menopausal symptoms (Messina, 2014), and a reduction in osteoporosis, breast cancer, and cardiovascular diseases (Michelfelder, 2009). This isoflavone is more selective for estrogen receptor (ER)/beta than estradiol which could explain such activities. The ingestion of soy isoflavones is considered safe, the most common adverse effects are gastrointestinal (diarrhea) and more rarely headache, prolonged menstrual period, amenorrhea, dizziness, and musculoskeletal complaints (Michelfelder, 2009). Some isoflavones, especially daidzin-daidzein, have been reported to inhibit the aldehyde-dehydrogenase-2 enzyme (ALDH2) (Gao et al., 2001; Lowe et al., 2008; Koppaka et al., 2012).

Kudzu (*Pueraria lobata*) root extracts and flowers contain isoflavones, for instance, puerarin (about 60% of the total isoflavones), and daidzin. Kudzu is used in traditional Japanese and Chinese medicine to treat alcohol hangover and alcohol substance use disorder (Overstreet et al., 2003a,b). Some studies in animal models have shown that daidzin and synthetic analogs have the capacity to reduce alcohol intake (Overstreet et al., 1996; Gao et al., 2003; Arolfo et al., 2009). In humans, a pilot study in a naturalistic setting demonstrated that the administration of a kudzu extract reduced alcohol consumption in heavy drinkers (Lukas et al., 2005). In controlled clinical trials with healthy volunteers, a reduction in alcohol intake when kudzu extract isoflavones were taken before drinking was reported (Penetar et al., 2012; Lukas et al., 2013; Penetar et al., 2015).

As already established, alcohol is biotransformed enzymatically to acetaldehyde by alcohol-dehydrogenase (ADH) and later metabolized into acetate by aldehyde-dehydrogenase (ALDH1). Disulfiram, used for the treatment of alcohol substance use, inhibits ALDH1 and ALDH2 irreversibly, producing an accumulation of acetaldehyde concentrations in blood which leads to undesirable effects if alcohol is simultaneously consumed. Symptoms, which include sickness, nausea, vomit, sweating, dizziness, and facial erythema, are known as the “disulfiram-reaction” or “antabuse-reaction.”

For centuries traditional Chinese medicine has employed different isoflavones for the treatment of alcohol substance use (Keung, 2003; Lu et al., 2009; Soyka and Rösner, 2010) with the advantage that they do not affect acetaldehyde levels, induce disulfiram-like effects (Penetar et al., 2011), or alter the sleep cycle (Bracken et al., 2011).

There are no previously published papers evaluating the possible interference/influence of soy isoflavone extracts on alcohol pharmacokinetics, acute pharmacological effects, and induction of a “disulfiram-like effect” despite their potential interaction in the treatment of alcohol abuse. The aim of the present study was to evaluate the interactions between a soy extract containing isoflavones and alcohol, in relation to the pharmacokinetics and acute effects of alcohol, in order to design future clinical trials to assess the possible therapeutic effects of isoflavones on substance use disorders, specifically cocaine.

## Materials and Methods

### Study Design

The study protocol was approved by the local Human Research Ethics Committee (CEIC-Parc de Salut Mar) and conducted in accordance with the Helsinki Declaration and local legislation. The trial was registered in a public database (ClinicalTrials.gov Identifier: NCT02309801). All the participants were informed about the study and signed a written informed consent before participation. All subjects were financially compensated for their participation.

The study was a randomized, single-blinded (the participants were informed that they could receive different doses of alcohol in the two sessions), and crossover clinical trial. Subjects participated in two different experimental sessions separated by at least 3 days. In one session they received a glass of water (240 mL) and 2 h later a single oral dose of alcohol was administered (0.5 g/kg, Vodka Absolut^®^ 40°, Sweden); in the other, they were given four capsules containing a soy extract product with a glass of water (240 mL) and 2 h later the same dose of alcohol.

### Soy Isoflavone Extract

A commercial soy extract product (Super-Absorbable Soy Isoflavones^®^, hard gelatin capsules, Life-Extension, United States) was employed. According to the manufacturer each capsule contained 54 mg of total isoflavones (22 mg daidzin-daidzein, 28 mg genistin-genistein, and 4 mg glycitin-glycitein). The dose administered (four capsules) was chosen in order to provide a total content of daidzin/daidzein (approximately 88 mg) which represented twice the dose recommended for the symptomatic therapy of menopausal symptoms (Brewer, 1984; Christensen et al., 1991; Gao et al., 2001) or that used in trials for asthma therapy (Smith et al., 2015).

### Subjects

A total of 10 healthy males were recruited from a volunteer database. The screening visit took place within the 3 weeks prior to commencing the study sessions. Subjects underwent a general physical examination, a 12-lead ECG, and a complete blood and urine analysis, including drugs of abuse in urine. Inclusion criteria were (i) recreational use of alcohol (<4 units of alcohol per day); (ii) previous experience of acute alcohol intoxication; and (iii) no history of substance abuse/dependence according to the Diagnosis and Statistical Criteria for Mental Disorders (DSM-IV-R). Exclusion criteria were (i) current tobacco smokers; (ii) current or previous history of mental disorders; (iii) any somatic illness that could interfere with alcohol and soy; (iv) the consumption of >5/day drinks of tea, coffee or similar xanthine-containing beverages during the 3 months prior to the study; and (v) the use of soy derivatives during the previous month.

### Procedure

Subjects were admitted to the Clinical Research Unit facilities at 07:45 a.m. after an overnight fast. Upon arrival, they were asked about any drug consumption or adverse event that could affect their participation. They were requested to refrain from using any psychoactive drug for a minimum of 7 days prior to the study and throughout it, and from consuming caffeinated products for 24 h and alcohol for 48 h. They were also asked to follow a diet free from daidzein (soy and derivatives) 3 days before and 48 h after each session. A urine sample was collected for drug of abuse testing (Instant-View^®^, Multipanel 10 Test Drug Screen, Alfa Scientific Designs Inc., Poway, CA, United States). Alcohol concentrations in breath were analyzed. An intravenous catheter in the non-dominant arm was inserted for blood sampling.

### Physiological and Subjective Effects

Non-invasive systolic blood pressure (SBP), diastolic blood pressure (DBP), heart rate (HR), oral and cutaneous facial temperature (OT, FT) were repeatedly recorded at baseline, immediately before capsule administration, and at 2 (immediately before beverage administration), 2.5, 3, 4, 5, 6, 8, and 10 h after first administration. All assessments were carried out with a DinamapTM 8100-T vital signs monitor (Critikon, Tampa, FL, United States). Cutaneous facial temperature was measured at the same time with an additional monitor (Critikon, Tampa, FL, United States).

Subjective effects were evaluated using a set of visual analog scales (VAS) at baseline (0 h), 2, 2.25, 2.5, 2.75, 3, 3.5, 4, 5, 6, 8, and 10 h after first administration. VAS (100 mm) were labeled with different adjectives marked at opposite ends from “not at all” to “extremely” (Peiró et al., 2013; Farré et al., 2015; Papaseit et al., 2016). Subjects were asked to rate effects of drunkenness, content, nausea, vertigo, dizziness, feeling of face flushing, headache, and breathing difficulty.

In addition, the 49-item short form Addiction Research Center Inventory (ARCI) was administered at baseline and at 2, 2.75, 3.5, and 10 h after first treatment administration. The Spanish validated version of the ARCI (Lamas et al., 1994) is sensitive to the effects of a variety of drug of abuse classes with five subscales: PCAG (pentobarbital-chlorpromazine-alcohol group, a measure of sedation); MBG (morphine-benzedrine group, a measure of euphoria); LSD (lysergic acid diethylamide group, a measure of dysphoria and somatic symptoms); BG (benzedrine group, a stimulant subscale relating to intellectual efficiency and energy); and A (amphetamine, a measure of d-amphetamine effects) (Farré et al., 2015).

### Pharmacokinetics

Blood samples and ethanol breath concentrations were collected at baseline (before soy extract administration), and at 2 (before ethanol administration), 2.25, 2.5, 2.75, 3, 4, 5, 6, 7, 8, 10, and 24 h after soy extract administration. Ethanol was determined in blood (DRI^®^ Ethyl Alcohol Assay, Thermo Fisher, Fremont, CA, United States) and breath (Alcotest, Dräger, Germany). Daidzein, genistein, and the endogenous metabolite equol were determined by liquid chromatography coupled to tandem mass spectrometry (LC/MS/MS) using a validated method (Rodríguez-Morató et al., 2015).

### Statistical Analysis

#### Effects

Differences with respect to baseline were calculated for vital signs (SBP, DBP, HR, and OT-FT) and subjective effects (VAS, ARCI). Maximum/peak effects (Emax) and the time to reach maximum effects (tmax) were also determined for the previously mentioned variables. The area under the curve of the concentrations from 0 to 10 h (AUC_0-10 h_) using the trapezoidal rule was calculated for vital signs and subjective effects.

For the statistical comparison of AUC and Emax a Student’s *t*-test for paired samples was employed. For tmax a non-parametric Wilcoxon test was used. A repeated measure ANOVA with two factors (treatment and time) was used to compare the time course of effects. When treatment or the treatment × time interaction was statistically significant, multiple Tukey *post hoc* comparisons were performed at each time point.

#### Experimental Pharmacokinetic Parameters

Peak concentration (Cmax), time to reach peak concentrations (Tmax), and area under the concentration-time curve from 0 to 10 h (AUC_0-10_) from ethanol plasma concentrations over time were determined using Pharmacokinetic Functions for Microsoft Excel (Joel Usansky, Atul Desai, and Diane Tang-Liu, Department of Pharmacokinetics and Drug Metabolism, Allergan, Irvine, CA, United States).

All statistical tests were performed with the PASW Statistics 18.0 (SPSS, Chicago, IL, United States). A value of *p* < 0.05 was considered statistically significant.

## Results

### Subject Characteristics

The 10 healthy male participants had a mean age of 25.2 ± 3.6 years, mean weight 74.0 ± 7.0 kg, and a body mass index of 23.3 ± 2.6. The participants consumed ethanol regularly (10.0 ± 7.1 standard drinks/week; 1 standard drink = 10 g of pure ethanol). All subjects completed the study. None required special therapy or care throughout the study and no serious adverse events occurred during the experimental sessions.

### Physiological Effects

**FIGURE 1:**
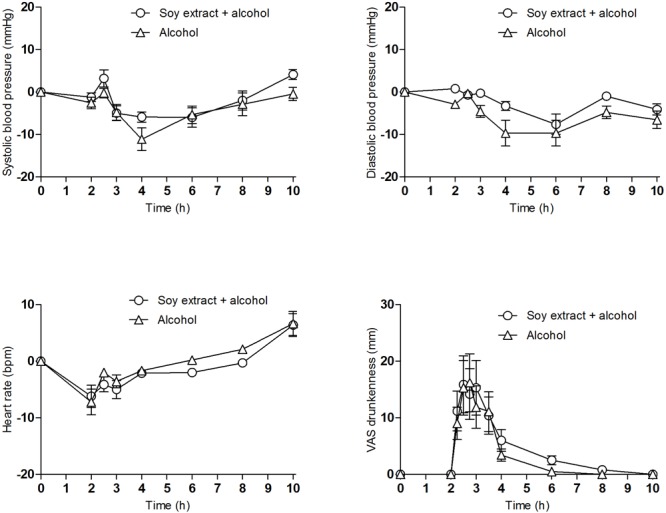
Time course of the physiological and subjective effects after administration of alcohol and soy extract+alcohol. Mean values and standard error (*n* = 10). Figures correspond to systolic and diastolic blood pressure (mmHg), heart rate (beats/min), and drunkenness (mm).

Table 1 shows a summary of the physiological and subjective effects. Regarding vital signs, no differences were observed in Emax and AUC for SBP, DBP, HR, and oral temperature between both conditions: alcohol and soy extract+alcohol (Figure 1, SBP, DBP HR). A slight difference in cutaneous facial temperature was found in the Emax (1.59 and 0.75°C after alcohol and soy extract+alcohol, respectively). In the time course analysis, a slightly higher reduction of DBP was reported at 2 h (-2.89, 0.8; *p* < 0.05), 3 h (-4.5, -0.3; *p* < 0.05), 4 h (-9.67, -3.3; *p* < 0.01), and 8 h (-4.78, -1; *p* < 0.05) after administration with alcohol alone in comparison with soy extract+alcohol, respectively.

**Table 1:** Summary of results of the physiological and subjective effects observed after administration of soy extract+alcohol and alcohol (*n* = 10).

Variable	Parameter	Soy extract+Alcohol	Alcohol	*P*-value
**Physiological**				
SBP	Emax	–4.5 ± 11.66	–9.2 ± 11.05	0.323
	AUC_0-10_	–24.4 ± 40.4	–39.17 ± 41.7	0.302
DBP	Emax	–4.8 ± 9.92	–10.5 ± 8.72	0.081
	AUC_0-10_	–25.8 ± 41.36	–58.8 ± 35.59	0.052
HR	Emax	–3.7 ± 13.09	–1.2 ± 15.26	0.368
	AUC_0-10_	–14.9 ± 61.2	–9.75 ± 59.58	0.759
OT	Emax	–0.26 ± 0.31	–0.23 ± 0.47	0.873
	AUC_0-10_	–0.967 ± 1.159	–1.2 ± 2.19	0.747
FT	Emax	0.75 ± 1.34	1.59 ± 0.67	**0.039**
	AUC_0-10_	6.04 ± 7.2	8.1 ± 5.59	0.205
**Visual Analog Scales**				
Drunkenness	Emax	17.6 ± 29.018	20.8 ± 19.1	0.602
	AUC_0-10_	35.36 ± 84.037	25.387 ± 35.1	0.569
Content	Emax	18.1 ± 30.2	23.9 ± 26.2	0.361
	AUC_0-10_	29.687 ± 67.9	29.91 ± 47.07	0.980
Nausea	Emax	0.5 ± 1.58	0.8 ± 1.75	0.718
	AUC_0-10_	0.2 ± 0.79	1.1 ± 3.1	0.416
Vertigo	Emax	3.2 ± 10.119	2.2 ± 6.9	0.812
	AUC_0-10_	5.27 ± 16.68	0.9 ± 3	0.449
Dizziness	Emax	9.4 ± 22.667	10.9 ± 18.3	0.687
	AUC_0-10_	19.9 ± 55.3	22.7 ± 47.09	0.627
Face flushing	Emax	7.5 ± 13.938	9.6 ± 14.9	0.696
	AUC_0-10_	16.3 ± 37.38	10.98 ± 20.8	0.614
Headache	Emax	5.3 ± 9.58	16.2 ± 27.9	0.217
	AUC_0-10_	18.16 ± 40.4	48.3 ± 87.9	0.274
Breathing difficulty	Emax	0	0	–
	AUC_0-10_	0	0	–
**ARCI questionnaire**				
ARCI-PCAG	Emax	2.4 ± 3.6	3.4 ± 3.59	0.266
	AUC_0-10_	9.8 ± 14.56	14.05 ± 14.2	0.237
ARCI-MBG	Emax	2.6 ± 2.836	2.7 ± 3.3	0.876
	AUC_0-10_	4.48 ± 5.01	3.4 ± 3.2	0.329
ARCI-LSD	Emax	4.8 ± 1.47	5.2 ± 1.7	0.373
	AUC_0-10_	42.26 ± 7.1	43.2 ± 8.27	0.328
ARCI-LSD	Emax	4.5 ± 0.97	4.6 ± 1.07	0.882
	AUC_0-10_	38.26 ± 5.8	36.7 ± 5.5	0.355
ARCI-BG ARCI-A	Emax	1.4 ± 1.4	1.8 ± 1.686	0.168
	AUC_0-10_	3.07 ± 3.8	4.5 ± 4.47	0.113

*Values are differences from baseline (mean values and standard deviation). Systolic blood pressure (SBP, mmHg); diastolic blood pressure (DBP, mmHg), heart rate (HR, beats/min), (temperature (T, °C), visual analog scale (VAS, mm), Addiction Research Center Inventory questionnaire (ARCI, score), Emax = maximal/peak effects, AUC_*0*-*10*_, area under the curve from 0 to 10 h.*

### Subjective Effects

In VAS, alcohol and soy extract+alcohol showed very similar subjective effects (drunkenness, content, nausea, vertigo, dizziness, face flushing, and breathing difficulty), without statistically significant differences (Figure 1, drunkenness). However, the alcohol alone condition, in contrast to the soy extract+alcohol, showed higher scores for headache at 8 h (16 mm, 3.7 mm, respectively, *p* < 0.01).

With regard to the ARCI questionnaire, no differences were reported between alcohol and soy extract+alcohol.

### Alcohol Concentrations

**FIGURE 2:**
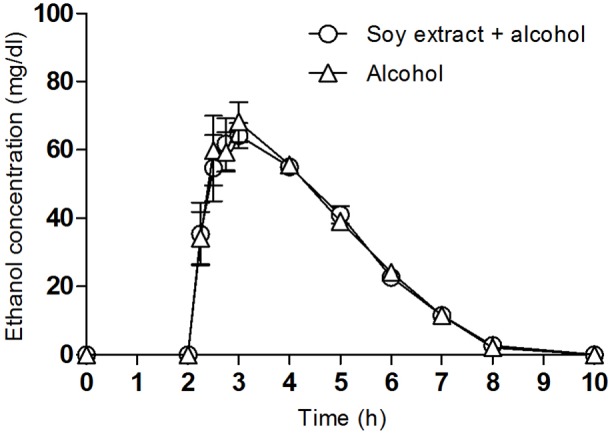
Plasma concentrations of ethanol after the administration of alcohol and soy extract + alcohol. Mean values and standard error (*n* = 10).

Pharmacokinetic parameters for blood ethanol concentrations over time curves are shown in Figure 2. No differences were observed for ethanol concentrations in blood between alcohol and soy extract+alcohol (Table 2). In both conditions, 10 h after drug administration, alcohol concentrations in plasma were deemed undetectable.

**Table 2:** Pharmacokinetic parameters of alcohol in plasma (*n* = 10).

Pharmacokinetic parameters	Soy extract + Alcohol	Alcohol	*P*-value
	Mean ± SD	Mean ± SD	
Cmax (ng/ml)	77 ± 16.99	77 ± 22.25	1
AUC_0-10_ (ng/ml/h)	216.57 ± 30.40	219.67 ± 27.2	0.806
Tmax (h)^∗^	2.75 (2.5-4)	3 (2.5-4)	0.256

*Mean values and standard deviation) (mean values and standard deviation) for Cmax and AUC. Median values and range for Tmax. AUC, area under the curve; Cmax, peak/maximal concentration; SD, standard deviation; Tmax, time to reach peak concentrations. ^∗^Median values and range for Tmax (Wilcoxon test).*

### Daidzein, Genistein, and Equol Concentrations

**FIGURE 3:**
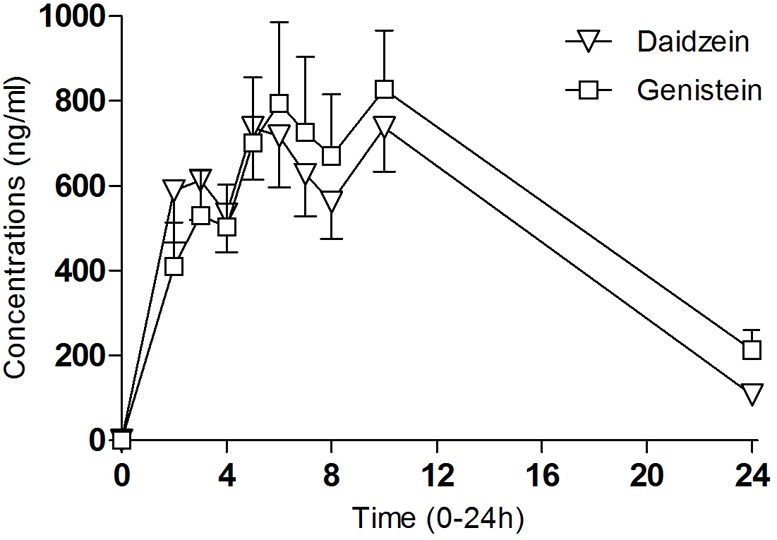
Plasma concentrations of daidzein and genistein after the administration of the soy extract. Mean values and standard error (*n* = 10).

Pharmacokinetic parameters for blood isoflavone concentrations (daidzein and genistein) over time curves are shown in Figure 3. Daidzein-concentrations peaked at 6.1 h (±3.2) after administration and genistein at 7.3 h (±3). The mean Cmax of daidzein was 1016.5 ng/ml (±303.1) and genistein 1039.0 ng/ml (±496.9). The concentrations of equol, a metabolite of daidzein, peaked at 24 h. Its pharmacokinetic parameters could not be calculated due to a limited number of evaluations (24 h). Multiple peaks along the plasma concentrations over time suggest an enterohepatic recirculation of isoflavones.

## Discussion

According to our findings, soy isoflavones neither interact with alcohol nor induce disulfiram-like effects with respect to alcohol pharmacokinetics and effects (vital signs and subjective/adverse effects). A previous study (Penetar et al., 2011) also suggested that the administration of a purified extract of a kudzu herbal medication containing isoflavones (mostly puerarin [19%], followed by daidzin [4%], and daidzein [2%]) did not increase the intoxicating effects of acute alcohol consumption in human volunteers (blood alcohol levels, subjective effects, and psychomotor performance).

We only found slightly significative changes in headache and, at some time points, facial temperature, and diastolic blood pressure when alcohol was administered. Such minimal changes could have been attributed to placebo (nocebo) effects, nevertheless, as a placebo capsule condition was not included we cannot substantiate this interpretation.

Our results were similar to a previous pharmacokinetic study in which the pharmacokinetic parameters for blood isoflavone concentrations showed double peaks in time-course plasma, thus supporting enterohepatic recirculation, characteristic of isoflavone metabolism (Rodríguez-Morató et al., 2015). We observed that the Tmax for alcohol appeared between 2.5 and 4 h after administration, the Tmax for the first peak for daidzein-genistein was between 2 and 4 h, and the maximal concentrations of both isoflavones were around 6 h. During 0.5–6 h blood alcohol levels were higher than those recommended for driving, within the range of the maximal isoflavone concentrations. As isoflavones show intensive presystemic metabolism concentrations in the liver they could be elevated enough at 0.5–2 h to inhibit hepatic metabolic enzymes (Chandrasekharan and Aglin, 2013; Rodríguez-Morató et al., 2015). We are, however, unaware of the consequences of alcohol administration regarding the time peak of isoflavone concentrations.

In contrast to disulfiram, some isoflavones are strong reversible inhibitors of ALDH2 and mild inhibitors of ALDH1. In the central nervous system, blocking ALDH2 inhibits dopamine metabolism by hindering the conversion of DOPAL to DOPAC, increasing levels of aldehyde DOPAL, and forming tetrahydropapaveroline (THP) in the ventral tegmental area. THP inhibits tyrosine hydroxylase (TH) and consequently decreases dopamine biosynthesis thus blunting the reinforcing effects of cocaine (Yao et al., 2010). In male mice, daidzein and genistein decreased cocaine-reinforcing effects, and daidzein, but not genistein, reduced cue-induced cocaine relapse (Martin et al., 2013). Alcohol is a substance commonly abused in combination with cocaine. A number of studies have found that about 60% of individuals with cocaine use disorder have co-occurring diagnoses of alcohol use disorder (Araos et al., 2017). Taking into account the pharmacodynamic profile of isoflavones, they could be used for future clinical trials studying possible treatments for substance use disorders (alcohol and cocaine,), without requiring absolute alcohol abstinence as an inclusion requirement.

Our study has some limitations. The sample included a small number of healthy volunteers and not patients with substance use disorders. Moreover, only males participated as isoflavones could present gender-based differences in metabolism (Soukup et al., 2016). Our research was performed using a single dose of isoflavones and a standard dose of alcohol for interaction studies. As the selected isoflavone dose was within the range of safe menopause symptom therapy, we are unable to advance results employing higher doses and/or repeated administrations. Disulfiram-like symptoms also appear at low-medium doses of alcohol when ALDH1 inhibitors are administered (Kline et al., 1987; Visapää et al., 2002). We employed a natural extract with a mixture of isoflavones and are thus unaware of whether the results could change after a single dose of each pure isoflavone or metabolite such as equol. Our study did not include a placebo condition, nevertheless, the non-significant symptomatic differences reported between the groups could be explained by placebo/nocebo effects. Neither were acetaldehyde concentrations analyzed nor any measure of psychomotor performance performed. The fact that Japanese and Chinese food contains elevated doses of isoflavones, and there are no adverse effects when alcohol is consumed, could support our results demonstrating a possible isoflavone mechanism of action on ALDH2. We did not, however, include a measure of the activity of these enzymes, and our findings do not provide significant evidence about the effect of soy extract on human aldehyde-dehydrogenase (or ALDH) isozymes.

## Conclusion

Substances such as soy isoflavones can be safely administered in combination with alcohol. They could be a new strategy for the treatment of substance use disorders without involving the risks associated with disulfiram.

## Data Availability

The datasets for this manuscript are not publicly available because Data will be available to other researchers following publication. Requests to access the datasets should be directed to mtorrens@parcdesalutmar.cat.

## Author Contributions

MF, MT, EP, CP-M, FF, and RM-R conceptualized the study design. MF, EP, CP-M, and RM-R collected the data. RdlT and NP analyzed the alcohol and isoflavones. MF, MT, EP, CP-M, FF, RdlT, and RM-R wrote the manuscript.

## Conflict of Interest Statement

The authors declare that the research was conducted in the absence of any commercial or financial relationships that could be construed as a potential conflict of interest.
